# Comparative genomic analysis of pre-epidemic and epidemic Zika virus strains for virological factors potentially associated with the rapidly expanding epidemic

**DOI:** 10.1038/emi.2016.48

**Published:** 2016-03-16

**Authors:** Zheng Zhu, Jasper Fuk-Woo Chan, Kah-Meng Tee, Garnet Kwan-Yue Choi, Susanna Kar-Pui Lau, Patrick Chiu-Yat Woo, Herman Tse, Kwok-Yung Yuen

**Affiliations:** 1 Department of Microbiology, The University of Hong Kong, Hong Kong, China; 2 State Key Laboratory of Emerging Infectious Diseases, The University of Hong Kong, Hong Kong, China; 3 Research Centre of Infection and Immunology, The University of Hong Kong, Hong Kong, China; 4 Carol Yu Centre for Infection, The University of Hong Kong, Hong Kong, China

**Keywords:** arbovirus, flavivirus, genome, microcephaly, mosquito, mutation, virus, Zika

## Abstract

Less than 20 sporadic cases of human Zika virus (ZIKV) infection were reported in Africa and Asia before 2007, but large outbreaks involving up to 73% of the populations on the Pacific islands have started since 2007, and spread to the Americas in 2014. Moreover, the clinical manifestation of ZIKV infection has apparently changed, as evident by increasing reports of neurological complications, such as Guillain–Barré syndrome in adults and congenital anomalies in neonates. We comprehensively compared the genome sequences of pre-epidemic and epidemic ZIKV strains with complete genome or complete polyprotein sequences available in GenBank. Besides the reported phylogenetic clustering of the epidemic strains with the Asian lineage, we found that the topology of phylogenetic tree of all coding regions is the same except that of the non-structural 2B (*NS2B*) coding region. This finding was confirmed by bootscan analysis and multiple sequence alignment, which suggested the presence of a fragment of genetic recombination at NS2B with that of Spondweni virus. Moreover, the representative epidemic strain possesses one large bulge of nine bases instead of an external loop on the first stem-loop structure at the 3′-untranslated region just distal to the stop codon of the NS5 in the 1947 pre-epidemic prototype strain. Fifteen amino acid substitutions are found in the epidemic strains when compared with the pre-epidemic strains. As mutations in other flaviviruses can be associated with changes in virulence, replication efficiency, antigenic epitopes and host tropism, further studies would be important to ascertain the biological significance of these genomic changes.

## INTRODUCTION

Zika virus (ZIKV) is an emerging mosquito-borne human-pathogenic flavivirus that has been mostly neglected due to its mild clinical manifestations and limited spread in restricted geographical regions in the first 60 years after its discovery.^1^ ZIKV was first isolated from the serum of a febrile sentinel rhesus macaque in 1947 in Zika Forest of Uganda.^2^ Between 1947 and 2006, <20 cases of human ZIKV infection were reported in the literature.^1^ These cases were geographically restricted in certain African (African lineage) and Southeast Asian (Asian lineage) countries. The first documented sizable outbreak of human ZIKV infection outside Africa and Asia occurred on Yap Island of the French States of Micronesia in 2007, during which 73% of the Yap population became infected.^3^ ZIKV then spread to other Pacific islands, and arrived in the western hemisphere in 2014 (Easter Island, Chile).^4, 5, 6^ Since then, many countries in the Americas have reported autochthonous cases of ZIKV infection. Brazil alone has reported an estimated 500 000–1 500 000 human cases of ZIKV infection in 2015.^7^ Although most patients with ZIKV infection are asymptomatic or have mild symptoms, life-threatening complications such as Guillain–Barré syndrome, thrombocytopenic purpura, and fatal disseminated disease in immunosuppressed hosts have been reported.^1, 3, 8^ Furthermore, preliminary epidemiological and virological data suggest that congenital ZIKV infection may be associated with microcephaly and other congenital anomalies in infected fetuses.^9, 10, 11^ The rapidly expanding epidemic and this suspected congenital ZIKV syndrome have led the World Health Organization to declare the ZIKV outbreak as a global public health emergency on 1 February 2016.^12^

The cause of the sudden emergence and rapid spread of ZIKV since 2007 is incompletely understood. A number of possible environmental factors have been proposed. First, globalization and urbanization have allowed ZIKV and its mosquito vectors to spread beyond their original geographical habitats. Second, major sport events including the World Cup and the Va'a World Sprint Championship canoe race in Brazil in 2014 might have provided an opportunity for infected travelers to introduce the virus to Latin America.^13^ Third, climate changes associated with El Niño in South America in 2015 on the background trend of global warming possibly facilitated the rapid spread of *Aedes* mosquitoes and ZIKV.^14^ Fourth, the increased awareness of and diagnostic capability for ZIKV infection likely led to the increased detection of this previously neglected disease. In contrast, little is known about the virological factors possibly associated with the apparent change in the spread of ZIKV after 2007. Although it has been shown that the epidemic strains are phylogenetically more closely related to the Asian than the African lineage of ZIKV, a comprehensive comparative analysis between the pre-epidemic and epidemic strains is lacking.^15^ In this study, we performed comparative genomic analysis of all the pre-epidemic and epidemic strains with complete genome or complete polyprotein sequences available in GenBank to identify possible viral factors associated with this rapidly emerging viral epidemic.

## MATERIALS AND METHODS

### Viral sequences

The genome sequences of 24 ZIKV isolates with complete genome or complete polyprotein sequences available in GenBank (accessed on 18 February 2016) were included in this study (Table 1). These included strains collected from human, animals, and mosquitoes in Africa, Asia, the Pacific islands, and Latin America between 1947 and 2015. Representative genome sequences of other human-pathogenic flaviviruses, including Spondweni virus (SPOV, DQ859064.1), dengue virus serotype 2 (DENV-2, NC_001474.2), Japanese encephalitis virus (JEV, NC_001437.1), West Nile virus (WNV, NC_001563.2), yellow fever virus (YFV, NC_002031.1) and tick-borne encephalitis virus (TBEV, NC_001672.1) were also included.

### Genomic characterization and phylogenetic analysis

Phylogenetic tree construction by the maximum likelihood method was performed using MEGA 6.0 software, with bootstrap values being calculated from 500 trees. Protein family analysis was performed using the PFAM tool (http://pfam.xfam.org) Search for Conserved Domains server (http://www.ncbi.nlm.nih.gov/Structure/cdd/wrpsb.cgi). Prediction of transmembrane domains was performed using the TMHMM 2.0 server (http://www.cbs.dtu.dk/services/TMHMM/). Prediction of signal peptides was performed by using signalP software 4.1 (http://www.cbs.dtu.dk/services/SignalP/). The prediction of potential O-glycosylation and N-glycosylation sites in the polyprotein was performed using NetOGlyc 4.0 (http://www.cbs.dtu.dk/services/NetOGlyc/) and NetNGlyc 1.0 (http://www.cbs.dtu.dk/services/NetNGlyc/), respectively. Secondary structure prediction in the 5′-untranslated region (UTR) was performed using the RNAfold WebServer with default settings (http://rna.tbi.univie.ac.at/cgi-bin/RNAfold.cgi). The number of synonymous substitutions per synonymous site, *Ks*, and the number of nonsynonymous substitutions per nonsynonymous site, *Ka*, for each coding region was calculated using the Nei and Gojobori substitution model with Jukes-Cantor correction in MEGA 6.0.^16, 17^ Bootscan analysis was performed using Simplot version 3.5.1 as described previously,^18^ with the Asian lineage of ZIKV strains as the query. Multiple alignment of the amino acid sequence of each protein are performed by ClustalX version 1.83 software and manually examined for any significant changes.

## RESULTS

### Genome arrangement

The single-stranded RNA genomes of the ZIKV strains used in this study range from 10 675 to 10 808 nucleotides encoding 3423 amino acids. The G+C content is 50.94%–51.26%. Similar to other flaviviruses, the ZIKV genomes have two flanking UTRs (5′- and 3′-UTR) and a single long open reading frame encoding a polyprotein. A type I cap structure without internal ribosomal entry site is present at the 5′ end followed by the conserved dinucleotide 5′-AG-3′. The 3′-end of the genome lacks a polyadenylate tail and terminates in a conserved 5′-CU-3′. The 5′- and 3′-UTRs are 107 and 429 nucleotides long, respectively. The 5′- and 3′-UTR sequences are conserved among ZIKV strains with nucleotide identities of ⩾83.6% and ⩾83.9%, respectively, but different from other flaviviruses including DENV, JEV, WNV, YFV, and TBEV (5′-UTR, ⩽69.5% 3′-UTR, ⩽63.4%).

**Figure 1:**
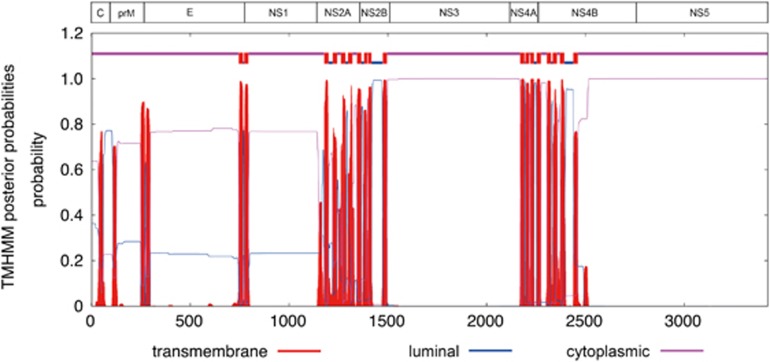
Putative transmembrane domains in the Zika virus genome. Abbreviations: capsid, C; envelope, E; nonstructural, NS; pre-Membrane, prM.

**Figure 2:**
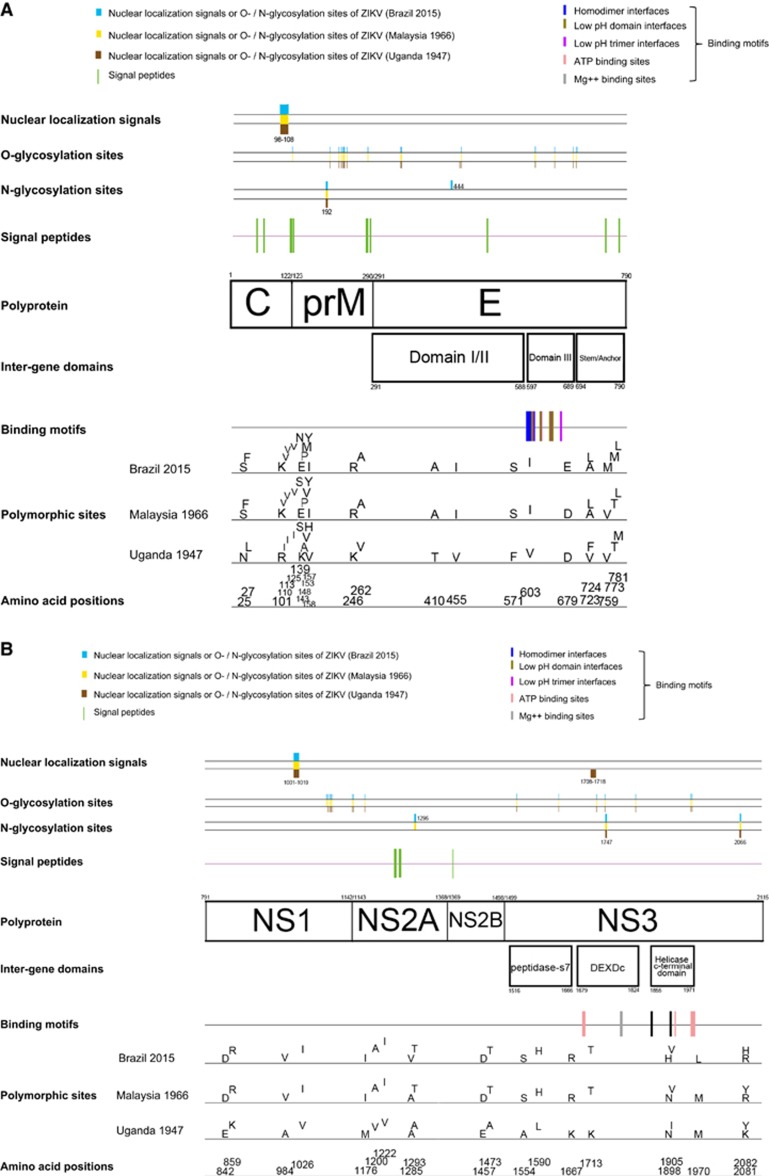

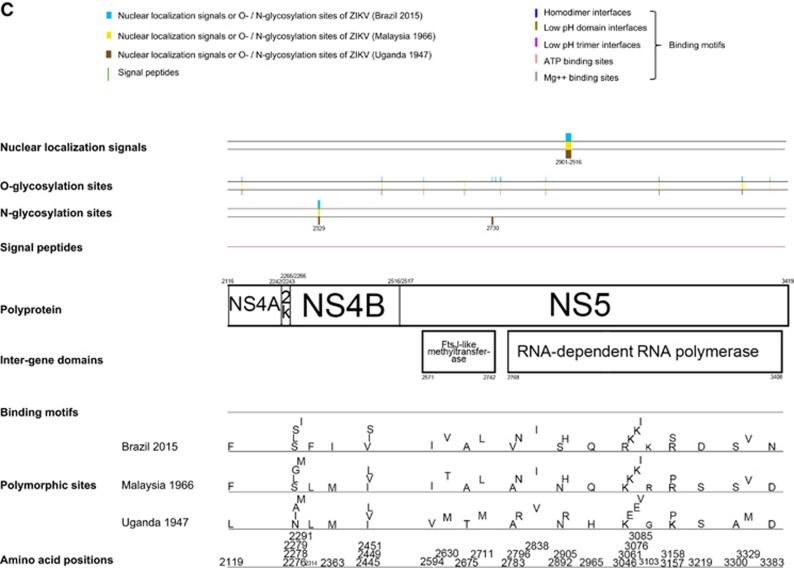
Comparative genomic analysis of the representative pre-epidemic and epidemic Zika virus strains. The results represent all 24 Zika virus strains unless otherwise specified (i.e., nuclear localization signals, and O-glycosylation and N-glycosylations sites). For the polymorphic sites, analysis was performed for all 24 Zika virus strains, but only the results of the three representative pre-epidemic African (Uganda 1947), pre-epidemic Asian (Malaysia 1966) and the epidemic (Brazil, 2015) strains are shown here. (**A**) Capsid (C), pre-Membrane (prM) and envelope (E); (**B**) nonstructural (NS) 1, NS2A, NS2B and NS3; (**C**) NS4A, NS4B and NS5. Abbreviation: DEAD-like helicase superfamily, DEXDc.

The polyprotein is cleaved into ten structural and non-structural (NS) proteins. Similar to other flaviviruses, the coding region orders and NS protein motifs of ZIKV are arranged in the order of 5′-Capsid (C)-preMembrane (prM)-Envelope (E)-NS1-NS2A-NS2B-NS3-NS4A-NS4B-NS5-3′. Cleavage at the N terminus of the signal sequence for NS4B generates a 23 amino acid peptide (the 2 K peptides at amino acid position 2243–2265). The complete polyprotein sequences of ZIKV have low similarity with those of other human-pathogenic flaviviruses (DENV-2, 58.1% to 58.9% SPOV, 68.3% to 69.0% nucleotide similarity). The ten structural and NS proteins contain multiple transmembrane domains that determine their location on the cytoplasmic or luminal side of the endoplasmic reticulum after cleavage (Figure 1). Most of the transmembrane domains are found in the NS2A, NS2B, NS4A, and NS4B proteins. This is compatible with the finding that the NS2A, NS2B, NS4A and NS4B proteins of other flaviviruses are mostly located within the endoplasmic reticular membrane bilayer, except for short regions between the transmembrane domains.^19^ The other NS proteins include NS1, a putative protease/helicase (NS3), and a putative RNA-dependent RNA polymerase (RdRp; NS5), which are essential components or enzymes involved in viral replication.^19^ Putative nuclear localization signals are found in C, NS1 and NS5 of both the pre-epidemic and epidemic strains, and NS3 of the prototype Uganda ZIKV strain (Figure 2). The putative O-glycosylation and N-glycosylation sites are mostly conserved, except for some variations at C, prM, E, NS1 and NS5 for O-glycosylation sites, and at E, NS2A and NS5 for N-glycosylation sites, which may be due to previous intracranial passage of the pre-epidemic Uganda and Malaysia ZIKV strains in mice.^20^

### Amino acid substitutions

Using all 24 available ZIKV genome sequences for analysis, the *Ka/Ks* ratios for the various coding regions were calculated (Table 2). Overall, the *Ka/Ks* ratios in ZIKV genomes are low, with the highest being observed at *C* coding region (0.077), suggesting that all the genes in the ZIKV genome are likely under stabilizing selection. Comparison between the pre-epidemic Asian lineage (Malaysia, 1966) and the epidemic ZIKV strains detected 24 amino acid substitutions (prM: 2, E: 3, NS2A: 1, NS3: 3, NS4B: 7 and NS5: 8) in the genomes of the latter virus strains (Figure 2). Four of these are associated with a change in the hydrophilicity/hydrophobicity of the amino acids (T773M in E, Y2082H in NS3, L2451S in NS4B and T2630V in NS5). Comparison between the pre-epidemic African lineage virus strains and the epidemic ZIKV strains detected 75 amino acid substitutions (C: 5, prM: 9, E: 10, NS1: 4, NS2A: 5, NS2B: 2, NS3: 9, NS4A: 1, NS4B: 9 and NS5: 21). Most of these are markers that differentiate the African and Asian lineages of ZIKV, which are also found in the pre-epidemic Asian lineage (Malaysia 1966) virus strain.^20^ Fifteen substitutions are only present in the epidemic ZIKV strains and not the pre-2007 strains (V153M in prM; D679E, V759M, and T773M in E; A1285V in NS2A; M1970L in NS3; L2314F, V2449I and L2451S in NS4B; A2783V, N2892S, K3046R, P3158S, S3219D, and D3383N in NS5).

### Phylogenetic relationship among all ZIKV strains

**Figure 3:**
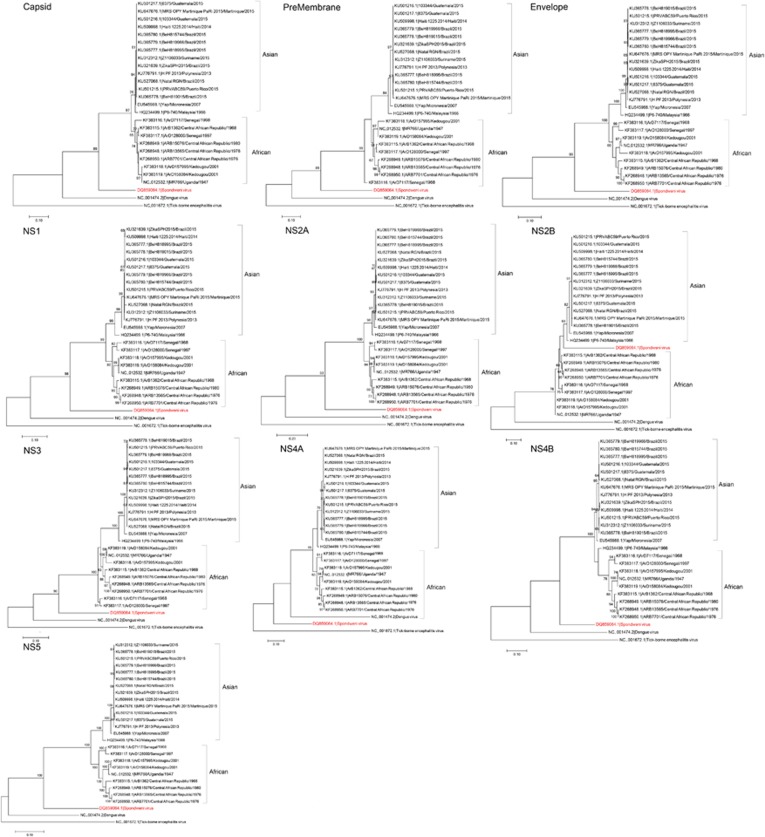
Phylogenetic analysis of the ten structural and non-structural coding regions of 24 Zika virus strains, rooted by Spondweni virus, dengue virus serotype 2, and tick-borne encephalitis virus. The trees were constructed by the maximum likelihood method based on the Tamura-Nei model. The tree with the highest log likelihood is shown. Initial tree(s) for the heuristic search were obtained automatically by applying Neighbor-Joining and BioNJ algorithms to a matrix of pairwise distances estimated using the Maximum Composite Likelihood approach, and then selecting the topology with superior log likelihood value. The bootstrap values were calculated from 500 trees. The tree is drawn to scale, with branch lengths measured in the number of substitutions per site. The bootstrap values <60% are not shown. All Zika virus strains are labeled as follow: accession number/strain number/country/year. All strains represent Zika virus strains unless otherwise specified for Spondweni virus, dengue virus serotype 2 and tick-borne encephalitis virus. Abbreviation: nonstructural, NS.

Phylogenetic analysis of the ten putative structural and *NS* coding regions showed that the ZIKV strains were clustered into the African and the Asian lineages (Figure 3). The epidemic ZIKV strains collected from the Pacific islands and South America clustered together with the Asian lineage strains. The complete polyprotein sequences of ZIKV within the same lineage (89.4%–99.8% nucleotide similarity) are more similar than those of different lineages (87.9%–95.5% nucleotide similarity). Notably, there is a change in the tree topology at the *NS2B* coding region, with a possible recombination occurring between ZIKV and SPOV.

### Recombination analysis

**Figure 4:**
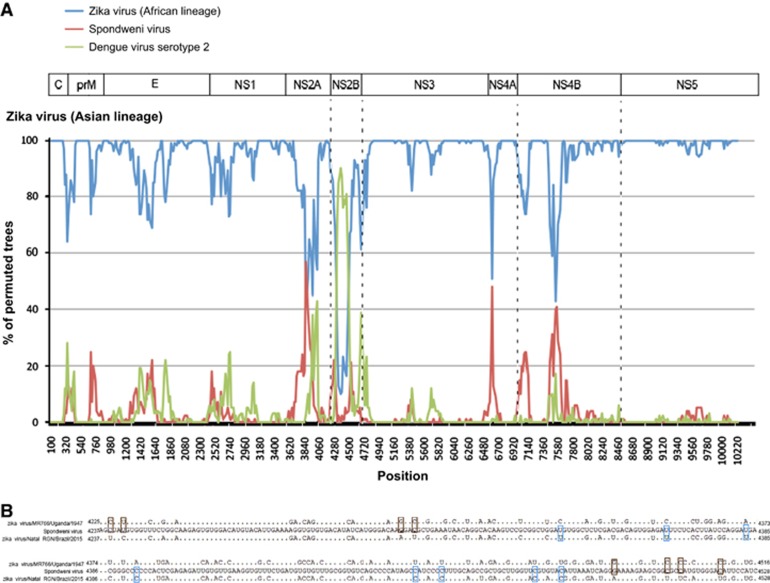
Genome organization and bootscan analysis of the Zika virus genomes. (**A**) Bootscanning was conducted with Simplot version 3.5.1 on a gapless nucleotide alignment, which was generated with ClustalX with the genome sequences of the available Asian lineage Zika virus strains as the query sequences. (**B**) Multiple alignment of the recombination fragment in NS2B nucleotide sequences of Zika virus strains MR766/Uganda/1947 (NC_012532.1) and Natal RGN/Brazil/2015 (KU527068.1), and Spondweni virus (DQ859064.1). In the Zika virus strains, only the nucleotides differing from those in Spondweni virus are depicted. The nucleotides in the Zika virus strains are highlighted in blue or red. Abbreviations: capsid, C; envelope, E; nonstructural, NS; pre-Membrane, prM.

In view of the change in tree topology at the *NS2B* coding region, we performed recombination analysis to look for potential recombination sites in the ZIKV strains. From the 5′-end to the 3′-end of the genome, bootscan analysis showed a possible recombination fragment from nucleotide positions 4237–4528 between the Asian lineage ZIKV strains and SPOV, when the genomes of Asian lineage of ZIKV strains were used as the query and the African lineage of ZIKV strains, SPOV and DENV-2 were used as the reference (Figures 4A and 4B). This finding correlates with the change in tree topology at the *NS2B* gene in the phylogenetic tree.

### RNA secondary structures and cyclization elements

**Figure 5:**
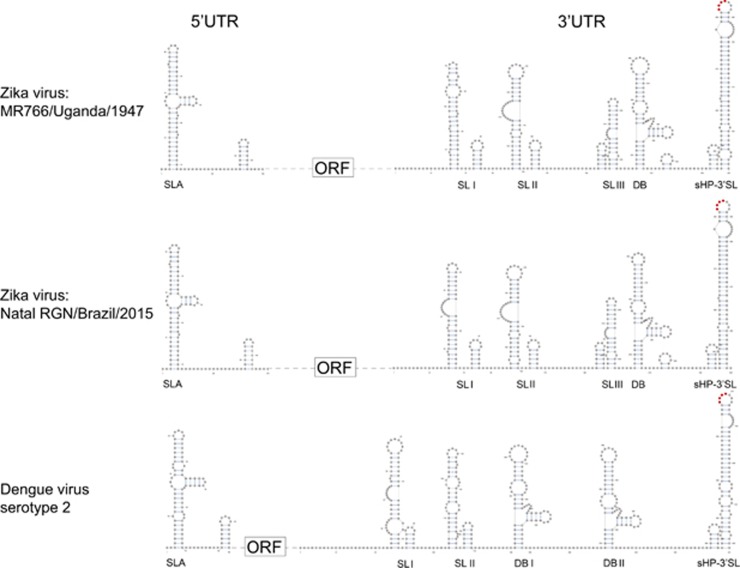
Schematic representations of the Zika virus genome RNA secondary structures. The short conserved 5′-ACAG-3′ sequences in the top loop of the sHP-3′ SL structure are indicated in red. Abbreviations: dumbbell, DB; open reading frame, sHP-3'-ORF; small hairpin 3'-stem-loop, sHP-3' SL; stem loop, SL; Y-shape stem-loop, SLA; untranslated region, UTR.

**Figure 6:**
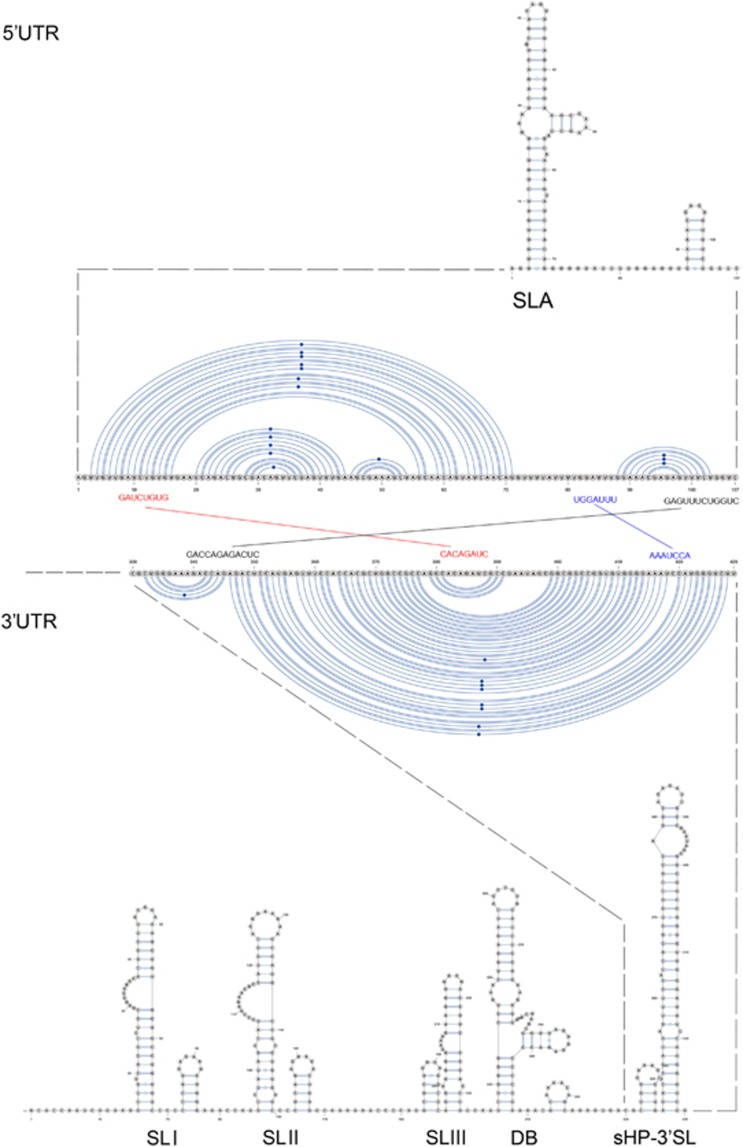
Terminal region genome sequences that are involved in 3′–5′ long distance RNA–RNA interactions. The three pairs of inverted complementary sequences that may mediate genome cyclization and allow the RdRp to reach the 3′ SL initiation site for RNA synthesis are enlarged. Abbreviations: dumbbell, DB; open reading frame, sHP-3' ORF; small hairpin 3'-stem-loop, sHP-3'-SL; stem Loop, SL; Y-shape stem-loop, SLA; untranslated region, UTR.

A Y-shape stem-loop A (SLA) structure is found at the 5′-end of the ZIKV genome (Figure 5). At the 3′-end of the viral genome, a small hairpin 3′-stem-loop (sHP-3′ SL) structure, three additional SL structures, and a dumbbell (DB) structure are found. Notably, the external loop of the SLI in domain 1 of the 3′-UTR just distal to the stop codon of the NS5 in the 1947 prototype pre-epidemic strain is replaced by a large bulge of nine nucleotide bases (UAG UCA GCC) in the representative epidemic ZIKV strain. Short conserved sequences within the 3′ terminal SL structure include the terminal 5′-CU-3′ and a 5′-ACAG-3′ in the top loop of the sHP-3′ SL structure. There are three pairs of inverted complementary sequences (GAU CUG UG-CAC AGA UC, UGG AUU U-AAA UCC A and GAG UUU CUG GUC-GAC CAG AGA CUC and GAG UUU CUG GUC-GAC CAG AGA CUC that may mediate genome cyclization (Figure 6).

## DISCUSSION

As demonstrated in recent epidemics of emerging viral infections, characterization of the viral genome may facilitate the identification of important virulence factors and diagnostic, therapeutic and vaccine targets.^21, 22, 23^ In this study, we analyzed the available genomic data of ZIKV in GenBank to provide a quick search for possible virus mutations that may be associated with the rapidly expanding ZIKV epidemic.

Our genomic analysis revealed some changes in the 3′-UTR sequence of the post-2007 epidemic ZIKV strain. The 5′ and 3′ terminal sequences of the genome of flaviviruses fold into conserved RNA secondary structures and encode regions essential for genome cyclization at the initial phase of replication.^24^ In mosquito-borne flaviviruses including ZIKV, the 3′ UTR is further divided into three domains, including the highly variable proximal domain 1 that directly follows the stop codon, the moderately conserved domain 2 that contains the SL and DB structures, and the highly conserved domain 3 that contains the complementary cyclization elements and the conserved sHP-3′ SL structure. Deletion of the SL sequences in the 5′- or 3′-UTR is lethal for flavivirus infectious clones.^25, 26^ These secondary RNA structures bind to host proteins, such as elongation factor 1α and poly(A)-binding protein, and proteins of the viral replication complex, including C, NS2A, NS3 and NS5 proteins, to promote genomic RNA cyclization.^27^ Genomic RNA cyclization is essential for viral replication in two ways. First, the 5′-SLA acts as a promotor element to stimulate the NS5 RdRp to initiate negative strand synthesis at the 3′-UTR.^28, 29, 30^ Second, the 5′- and 3′-UTRs move into close proximity for cap-dependent translation of the viral polyprotein to proceed.^31, 32, 33^

The two most conserved secondary RNA structures in flavivirus genomes are the Y-shape SLA structure at the 5′-UTR and the sHP-3′ SL structure at the 3′-UTR.^24^ Expectedly, these are also present in the ZIKV genomes. The arrangement and sequences of the other 3′-UTR RNA secondary structures of ZIKV are less conserved from those of other flaviviruses.^24^ For example, there are two DB structures and two SL structures in addition to the conserved sHP-3′ SL structure in DENV-1 and DENV-3, whereas three additional putative SL and one DB structures are found in both the pre-epidemic and epidemic ZIKV strains.^24^ Interestingly, we found a large bulge of nine nucleotide bases at the SLI of the epidemic ZIKV strain, which more closely resembles the SLII than the corresponding SLI of the pre-epidemic strain. Wet laboratory experiments are required to investigate the functional role of this putative conformational change in the transmissibility and virulence of the epidemic ZIKV strain.

After cleavage of the polyprotein, the C protein of flaviviruses is released into the cytoplasm and forms homodimers. The basic residues on one side of the C protein bind the RNA genome and the hydrophobic residues on the other side interact with the viral lipid envelope.^34, 35^ Even after virus-endosomal membrane fusion, the entering viral genome may remain associated with the C dimers to evade from host nucleases and RNA sensors. Thus, the C protein of flaviviruses may function as an RNA chaperone in addition to its role in the formation of viral nucleocapsid. The resulting nucleocapsid then buds into the endoplasmic reticular lumen to form viral particles with the prM and E proteins.^36, 37^ The C protein may also be found in the nuclei and nucleoli of cell lines infected by flaviviruses, including DENV-2, DENV-4, WNV, JEV and Kunjin virus.^38, 39, 40^ The migration of the C protein to the nuclei and nucleoli are believed to be mediated by nuclear localization signals.^41^ In ZIKV, we found one putative nuclear localization signal near the 3′-end of the C protein, which is conserved in the pre-epidemic and epidemic strains (Figure 2A). JEV with a single point mutation (T45G) at the N terminus of the *C* coding region has reduced virulence.^42^ In our study, we found five amino acid substitutions in C protein including N25S, L27F, R101K, I110V and I113V, which were detected in the Asian ZIKV strains as compared with the prototype Uganda African ZIKV strain. The importance of these findings should be verified in future experiments.

The prM protein of flaviviruses interacts with the E protein to form prM-E heterodimers, which are essential for the formation of immature virions. The prM protein is then cleaved to M protein through cellular proteases to produce and release mature virions. The M protein of flaviviruses contains two membrane-spanning domains and a short ectodomain. Histidine at residue 99 and the transmembrane region of the prM protein of JEV is critical for stable prM-E heterodimeric complex formation.^43^ Moreover, a single amino acid substitution at the N-linked glycosylation site of the prM-E complex of JEV may elicit an enhanced host humoral immune response, which could be a useful strategy for vaccine design.^44^ The functional roles of the V153M substitution, which is found in all the ZIKV epidemic strains, and other amino acid substitutions I125V, S139N, K143E, A148P, H157Y, V158I, K246R and V262A, which are different between the African and Asian lineages, may be of some importance in future studies.

The E protein is the major surface protein of flaviviruses and is involved in viral attachment, fusion, penetration, hemagglutination, host range and cell tropism.^45^ Structurally, three domains could be found in the E protein of flaviviruses based on X-ray crystallographic structural studies. Domain I is located in the middle of the E protein and contains the N terminus with glycosylation sites. Domain II contains the fusion peptide at the distal side and flanks one side of domain I. Domain III flanks the other side of domain I and is the major antigenic region in the E protein. It also contains the receptor-binding site and is, therefore, an important therapeutic and vaccine target of flaviviruses. E345K substitution at the E protein of DENV-4 is associated with reduced viral virulence.^46^ Importantly, we found a number of amino acid substitutions in the E protein of the epidemic ZIKV strain (Figure 2A). Among these substitutions, V603I and D679E are found in the domain III of the E protein. The I (isoleucine) at position 603 and the E (glutamic acid) at position 679 are present in all of the epidemic strains, but in none of the pre-epidemic strains. Investigation on the presence and function of these amino acid substitutions should be performed on a larger collection of epidemic strains.

NS1 of flaviviruses is a glycoprotein that may contain multiple N-glycosylation sites and disulfide bonds that may affect virus viability and virulence.^47^ Significant NS1 codon usage adaptation to human housekeeping genes by the recent Asian lineage of ZIKV has been suggested to be a facilitator of viral replication and increased viral titers.^48^ NS1 may exist in different forms, depending on the variable formation of N-glycosylation sites and disulfide bonds. The monomers of NS1 are soluble and hydrophilic, whereas the NS1 homodimers may associate with the endoplasmic reticular membranes.^49, 50^ The NS1 protein also exists in a soluble hexamer form that is secreted by mammalian cells.^19^ Mutations at the NS1 N-glycosylation sites may significantly affect viral replication and virulence in YFV.^51^ Deletion studies suggest that NS1 is required for initiation of RNA synthesis and especially during early negative strand RNA genome synthesis.^19^ Using the available genomic data in this study, we were not able to find any putative N-glycosylation sites in NS1 of the ZIKV genomes, but we found four different amino acid substitutions between the African and the Asian lineages (E842D, K859R, A984V and V1026I). The importance of these substitutions will await further verifications.

NS2A, NS2B, NS4A and NS4B of flaviviruses are small, hydrophobic proteins with incompletely understood functions and no known enzymatic motifs.^19^ Each of these proteins has two or more membrane-spanning regions and may play important roles in the assembly or anchoring of the viral replication complexes on the endoplasmic reticular membrane.^52, 53^ Mutation at the helix-breaker amino acid R84 at the NS2A of WNV may attenuate viral replication.^19^ NS2B interacts with the NS3 C-terminal protease domain to form the viral serine protease complex that is involved in the cleavage of the viral polyprotein. Moreover, these NS proteins may exert important effects on the host immune response. The DENV NS2B/NS3 complex has been shown to mediate cleavage of STING, which is a key mediator in the pathways of the host innate immune response.^54^ Expression of WNV and Kunjin virus NS2A, NS2B, NS4A and NS4B proteins may also block type I interferon signaling.^55, 56^ Mutations at the NS4B of WNV may attenuate the neurovirulence and viral replication in infected mice.^57, 58^ We found two amino acid substitutions that are present in all the epidemic strains, including V2449I and L2451S, among the analyzed ZIKV strains (Figure 2C). Interestingly, we also found that there is likely a recombination between the NS2B of ZIKV and SPOV, although the direction of gene transfer was uncertain (Figures 3 and 4). A recent study did not detect recombination events between the epidemic Brazilian ZIKV strain and other arboviruses including DENV-1, DENV-4, WNV, YFV and Chikungunya virus, but SPOV was not included in the analysis.^10^ Further investigations are necessary to determine the biological significance of these amino acid substitutions among the ZIKV strains and this recombination between the two human-pathogenic mosquito-borne flaviviruses.

The major viral enzymes of flaviviruses are encoded by the *NS3* and *NS5* coding regions. In addition to its putative protease activity at the N terminus, the C-terminal domain of the NS3 protein of ZIKV also possesses putative ATPase/helicase, nucleoside triphosphatase, and 5′-triphosphatase activities. The multiple roles of NS3 in the viral replication cycle make the protein an attractive antiviral target. NS5 is the largest and most conserved protein of ZIKV. The N-terminal part contains a putative methyltransferase domain with both N7 and 2′-*O*-methyltransferase and guanyltransferase activities. The C-terminal part contains typical motifs of RdRp as in other flaviviruses. The NS5 protein of DENV mediates cleavage of STAT2, which is an important mediator in the host innate immune response signaling pathways.^59^ In this study, we found that NS5, being the largest protein, also has the largest number of amino acid substitutions (*n*=21) when the strains of African and Asian lineages are compared. Eight amino acid substitutions in the NS3 and NS5 are different between the pre-epidemic and epidemic strains, including M1970L, T2630V, A2783V, N2892S, K3046R, P3158S, S3219D and D3383N. The importance of these substitutions should be investigated further.

Although ZIKV was estimated to have emerged between 1892 and 1943 by Bayesian evolutionary analysis and has been isolated for nearly 70 years, very little is known about the virology of this emerging virus.^60^ In this study, we summarized its genomic changes with a limited number of virus strains. These virus strains included both the pre-epidemic African and Asian lineage strains that were mostly mosquito isolates, and the epidemic strains found in human. Among these virus strains, we have detected a number of amino acid substitutions throughout the genome and a conformational change in the SLI structure at the 3′-UTR of the epidemic ZIKV strain. We have also detected a possible recombination of a *NS2B* fragment between the Asian lineage of ZIKV and SPOV. The impact of these changes of the virus genome on the virulence, viability and transmissibility of ZIKV should be further investigated in biological assays.

## Figures and Tables

**Table 1:** Genome sequences of Zika virus strains in this study

**Accession number**	**Virus strain**	**Year**	**Country**	**Species/Source**
**Complete genome**				
NC_012532.1	MR766/Uganda/1947	1947	Uganda	*Macaca mulatta*
KU509998.1	Haiti 1225 2014/Haiti/2014	2014	Haiti	*Homo sapiens*
KU321639.1	ZikaSPH2015/Brazil/2015	2015	Brazil	*Homo sapiens*
KU501215.1	PRVABC59/Puerto_Rico/2015	2015	Puerto Rico	*Homo sapiens*
KU365780.1	BeH815744/Brazil/2015	2015	Brazil	*Homo sapiens*
				
**Complete polyprotein**				
HQ234499.1	P6-740/Malaysia/1966	1966	Malaysia	*Aedes aegypti*
KF383115.1	ArB1362/Central African Republic/1968	1968	CAP	*Aedes africanus*
KF268950.1	ARB7701/Central African Republic/1976	1976	CAP	*Aedes africanus*
KF268948.1	ARB13565/Central African Republic/1976	1976	CAP	*Aedes africanus*
KF268949.1	ARB15076/Central African Republic/1980	1980	CAP	*Aedes opok*
KF383116.1	ArD7117/Senegal/1968	1968	Senegal	*Aedes luteocephalus*
KF383117.1	ArD128000/Senegal/1997	1997	Senegal	*Aedes luteocephalus*
KF383118.1	ArD157995/Kedougou/2001	2001	Kedougou	*Aedes dalzieli*
KF383119.1	ArD158084/Kedougou/2001	2001	Kedougou	*Aedes dalzieli*
EU545988.1	Yap/Micronesia/2007	2007	Micronesia	*Homo sapiens*
KJ776791.1	H PF 2013/Polynesia/2013	2013	Polynesia	*Homo sapiens*
KU365777.1	BeH818995/Brazil/2015	2015	Brazil	*Homo sapiens*
KU365778.1	BeH819015/Brazil/2015	2015	Brazil	*Homo sapiens*
KU365779.1	BeH819966/Brazil/2015	2015	Brazil	*Homo sapiens*
KU501216.1	103344/Guatemala/2015	2015	Guatemala	*Homo sapiens*
KU501217.1	8375/Guatemala/2015	2015	Guatemala	*Homo sapiens*
KU647676.1	MRS OPY Martinique PaRi 2015/Martinique/2015	2015	Martinique	*Homo sapiens*
KU312312.1	Z1106033/Suriname/2015	2015	Suriname	*Homo sapiens*
KU527068.1	Natal RGN/Brazil/2015	2015	Brazil	*Homo sapiens*

Abbreviation: Central African Republic, CAP.

**Table 2:** Estimation of nonsynonymous and synonymous substitution rates in the 24 ZIKV genome sequences

**Coding region**	**Nucleotide position**^a^	**Amino acid position**^a^	**Substitution rate**		
			***Ka***	***Ks***	***Ka/Ks***
Polyprotein	1–10257	1–3419	0.010	0.362	0.028
Capsid	1–366	1–122	0.016	0.209	0.077
Pre-membrane	367–870	123–290	0.018	0.366	0.049
Envelope	871–2370	291–790	0.008	0.388	0.021
NS1	2371–3426	791–1142	0.006	0.372	0.016
NS2A	3427–4104	1143–1368	0.013	0.360	0.036
NS2B	4105–4494	1369–1498	0.003	0.333	0.009
NS3	4495–6345	1499–2115	0.008	0.385	0.021
NS4A	6346–6726	2116–2242	0.003	0.347	0.009
NS4B	6796–7548	2266–2516	0.011	0.338	0.033
NS5	7549–10257	2517–3419	0.012	0.413	0.029

a The positions refer to the first nucleotide or amino acid position in the polyprotein of the ZIKV strain MR766/Uganda/1947 (NC_012532.1). The nucleotide position of the polyprotein and NS5 coding region does not include the stop codon at the *NS5* coding region.
